# Physics of the Infections Diseases

**Published:** 1878-08

**Authors:** 


*Physics of the Infections Diseases.* By C. A. Logan, A. M., M. D. Chicago: Jansen McClarg & Co. 12mo. pp. 212.

An experience as a member of the Kansas State Geological Survey, In charge of the department of Botany and Sanitary relations, and a residence in South America, as United States Minister to Chili, has led the author to observe that the prevalence of certain acute infectious diseases is associated with certain general physical conditions. His desire to direct attention to this subject and to make record of the peculiar physical conditions and sanitary influence of the climate of the volcanic regions of South America, has led to the writing of this volume. The phenomena attending earthquakes, the effect of rains, ozone investigations, prevalent diseases and their origin, are topics that are attractive to the reader. The writer states that vaccination in Chlli has proved inefficacious in preventing small pox and that repeated attacks of this disease have been experienced by many individuals there.

The book will be interesting to readers of sanitary literature.

